# Supplementary material for: Concerted action of the MutLβ heterodimer and Mer3 helicase regulates the global extent of meiotic gene conversion
Source: eLife. 2017 Jan 4;6:e21900. doi: 10.7554/eLife.21900 (PMC5215242; doi:10.7554/eLife.21900)
Supplement: Suplementary file 1. — DOI: http://dx.doi.org/10.7554/eLife.21900.021 [file elife-21900-fig1.docx]

**Supplementary File 1: Spore viabilities**

| Strain name | Genotype | Tetrade type (%) | | | | | Spore viability (%) | Number of tetrads | Fisher Test p-Value |
| --- | --- | --- | --- | --- | --- | --- | --- | --- | --- |
|  |  | 4 sp | 3 sp | 2 sp | 1 sp | 0 sp |  |  |  |
| VBD1311 | WT | 89.1 | 6.1 | 4.8 | 0.0 | 0.0 | 96.1 | 147 | vs WT |
| VBD1631 | *mlh2∆* | 92.2 | 3.3 | 4.4 | 0.0 | 0.0 | 96.9 | 90 | 0.59 |
| VBD1635 | *mer3R893E* | 86.7 | 5.6 | 7.0 | 0.0 | 0.7 | 94.4 | 143 | 0.21 |
| VBD1750 | *mer3-hd* | 70.8 | 6.9 | 11.1 | 4.2 | 6.9 | 82.6 | 72 | 1.2E-10 |
| VBD1756 | *mer3-hd mlh2∆* | 73.1 | 10.3 | 11.5 | 1.3 | 3.8 | 86.9 | 78 | 8.8E-07 |
| VBD1082 | *zip4∆* | 27.3 | 4.7 | 16.4 | 7.0 | 44.5 | 40.8 | 128 | vs *zip4∆* |
| VBD1602 | *mlh2∆ zip4∆* | 33.8 | 4.6 | 25.4 | 5.4 | 30.8 | 51.3 | 130 | 7.5E-04 |
| VBD1653 | *mer3R893E zip4∆* | 30.0 | 11.8 | 20.0 | 5.5 | 32.7 | 50.2 | 110 | 4.1E-03 |
| VBD1649 | *MLH2-Myc zip4∆* | 22.9 | 4.2 | 22.9 | 10.4 | 39.6 | 40.1 | 48 | 0.9 |
| VBD1676 | *msh4∆* | 22.5 | 2.5 | 20.0 | 2.5 | 52.5 | 35.0 | 120 | vs *msh4∆* |
| VBD1682 | *mlh2∆ msh4∆* | 35.0 | 5.0 | 29.0 | 2.0 | 29.0 | 53.8 | 100 | 2.9E-08 |
| VBD1684 | *mer3R893E msh4∆* | 24.8 | 10.6 | 26.2 | 11.3 | 27.0 | 48.8 | 141 | 8.0E-06 |
| VBD1414 | *mer3∆* | 18.5 | 2.3 | 22.3 | 10.8 | 46.2 | 34.0 | 130 | vs *mer3∆* |
| VBD1604 | *mlh2∆ mer3∆* | 12.8 | 4.5 | 16.7 | 1.9 | 64.1 | 25.0 | 156 | 8.6E-04 |
| VBD1726 | *mer3Δ zip4∆* | 17.4 | 6.6 | 17.4 | 6.6 | 52.1 | 32.6 | 121 | vs *mer3Δ zip4∆* |
| VBD1714 | *mlh2∆ mer3Δ zip4∆* | 8.5 | 3.0 | 12.2 | 6.1 | 70.1 | 18.4 | 164 | 4.8E-08 |
| VBD1757 | *mer3-hd* *msh4∆* | 26.7 | 3.5 | 22.1 | 7.0 | 40.7 | 42.2 | 86 | *vs mer3-hd msh4∆* |
| VBD1758 | *mer3-hd* *msh4∆* *mlh2∆* | 46.2 | 3.8 | 19.2 | 5.1 | 25.6 | 59.9 | 78 | 5.7E-06 |
| HY1 | *msh2∆* | 40.0 | 27.5 | 21.7 | 10.8 | 0.0 | 74.2 | 120 | vs *msh2∆* |
| HY2 | *msh2∆ mlh2∆* | 18.9 | 28.7 | 28.7 | 13.9 | 9.8 | 58.2 | 122 | 1.6E-07 |
| HY3 | *msh2∆ mer3R893E* | 15.6 | 35.4 | 27.1 | 17.7 | 4.2 | 60.2 | 96 | 1.4E-05 |
| HY4 | *msh2∆ mer3-hd* | 35.8 | 22.5 | 30.0 | 8.3 | 3.3 | 69.8 | 120 | 0.15 |
| HY5 | *msh2∆ mer3-hd mlh2∆* | 20.1 | 20.8 | 20.1 | 21.5 | 17.4 | 51.2 | 144 | 1.7E-14 |
| HY6 | WT | 42.3 | 32.1 | 23.8 | 1.8 | 0.0 | 78.7 | 168 | vs WT |
| HY7 | *mlh2∆* | 31.0 | 31.0 | 25.6 | 9.5 | 3.0 | 69.3 | 168 | 1.1E-04 |
