# Supplementary material for: Concerted action of the MutLβ heterodimer and Mer3 helicase regulates the global extent of meiotic gene conversion
Source: eLife. 2017 Jan 4;6:e21900. doi: 10.7554/eLife.21900 (PMC5215242; doi:10.7554/eLife.21900)
Supplement: Supplementary file 2. — DOI: http://dx.doi.org/10.7554/eLife.21900.022 [file elife-21900-supp2.docx]

**Supplementary File 2: Genotypes of strains used in this study.**

Diploids : The MATa parent is indicated first

**Strain name Genotype**

VBD1082 *a/l ho::hisG/" leu2::hisG/’’ ura3/'' HIS4::LEU2-(BamH1; +ori)/his4-X::LEU2-(NgoMIV; +ori)-URA3 zip4∆::HphMX/”*

VBD1311 *a/l ho::hisG/" leu2::hisG/’’ ura3/'' HIS4::LEU2-(BamH1; +ori)/his4-X::LEU2-(NgoMIV; +ori)-URA3*

VBD1382 *a/l ho::hisG/" leu2::hisG/’’ ura3/'' HIS4::LEU2-(BamH1; +ori)/his4-X::LEU2-(NgoMIV; +ori)-URA3 spo11∆::HphMX/”*

VBD1337 *a/l ho::hisG/" leu2::hisG/’’ ura3/'' HIS4::LEU2-(BamH1; +ori)/his4-X::LEU2-(NgoMIV; +ori)-URA3 MLH1-710-His6-Flag3-713::KanMX/''*

VBD1414 *a/l ho::hisG/" leu2::hisG/’’ ura3/'' HIS4::LEU2-(BamH1; +ori)/his4-X::LEU2-(NgoMIV; +ori)-URA3 mer3∆::HphMX/”*

VBD1420 *a/l ho::hisG/" leu2::hisG/’’ ura3/'' HIS4::LEU2-(BamH1; +ori)/his4-X::LEU2-(NgoMIV; +ori)-URA3 MER3-His6-Flag3::NatMX/''*

VBD1454 *a/l ho::hisG/" leu2::hisG/’’ ura3/'' HIS4::LEU2-(BamH1; +ori)/his4-X::LEU2-(NgoMIV; +ori)-URA3 MLH1-710-HA6-713::KanMX/'' MER3-His6-Flag3 ::NatMX/''*

VBD1456 *a/l ho::hisG/" leu2::hisG/’’ ura3/'' HIS4::LEU2-(BamH1; +ori)/his4-X::LEU2-(NgoMIV; +ori)-URA3 MLH1-710-HA6-713::KanMX/''*

VBD1490 *a/l ho::hisG/" leu2::hisG/’’ ura3/'' HIS4::LEU2-(BamH1; +ori)/his4-X::LEU2-(NgoMIV; +ori)-URA3 MLH1-710-HA6-713::KanMX/'' MER3-1-976-His6-Flag3::NatMX/''*

VBD1494 *a/l ho::hisG/" leu2::hisG/’’ ura3/'' HIS4::LEU2-(BamH1; +ori)/his4-X::LEU2-(NgoMIV; +ori)-URA3 mlh1∆::HphMX/”*

VBD1550 *a/l ho::hisG/" leu2::hisG/’’ ura3/'' HIS4::LEU2-(BamH1; +ori)/his4-X::LEU2-(NgoMIV; +ori)-URA3 MLH1-710-HA6-713::KanMX/'' MER3-His6-Flag3::NatMX/'' mlh2∆::HphMX/”*

VBD1552 *a/l ho::hisG/" leu2::hisG/’’ ura3/'' HIS4::LEU2-(BamH1; +ori)/his4-X::LEU2-(NgoMIV; +ori)-URA3 MLH1-710-HA6-713::KanMX/'' MER3-1-976-His6-Flag3::NatMX/'' mlh2∆::HphMX/”*

VBD1564 *a/l ho::hisG/" leu2::hisG/’’ ura3/'' HIS4::LEU2-(BamH1; +ori)/his4-X::LEU2-(NgoMIV; +ori)-URA3 MLH1-710-HA6-713::KanMX/'' mer3∆::HphMX/MER3*

VBD1576 *a/l ho::hisG/" leu2::hisG/’’ ura3/'' HIS4::LEU2-(BamH1; +ori)/his4-X::LEU2-(NgoMIV; +ori)-URA3 MLH1-710-HA6-713::KanMX/'' mer3∆::HphMX/MER3-His6-Flag3::NatMX*

VBD1579 *a/l ho::hisG/" leu2::hisG/’’ ura3/'' HIS4::LEU2-(BamH1; +ori)/his4-X::LEU2-(NgoMIV; +ori)-URA3 MLH1-710-HA6-713::KanMX/'' mer3∆::HphMX/MER3∆836-941-His6-Flag3::NatMX/''*

VBD1602 *a/l ho::hisG/" leu2::hisG/’’ ura3/'' HIS4::LEU2-(BamH1; +ori)/his4-X::LEU2-(NgoMIV; +ori)-URA3 mlh2∆::HphMX/” zip4∆::HphMX/”*

VBD1604 *a/l ho::hisG/" leu2::hisG/’’ ura3/'' HIS4::LEU2-(BamH1; +ori)/his4-X::LEU2-(NgoMIV; +ori)-URA3 mlh2∆::HphMX/” mer3∆::HphMX/”*

VBD1628 *a/l ho::hisG/" leu2::hisG/’’ ura3/'' HIS4::LEU2-(BamH1; +ori)/his4-X::LEU2-(NgoMIV; +ori)-URA3 MLH2-Myc13::HphMX/”*

VBD1629 *a/l ho::hisG/" leu2::hisG/’’ ura3/'' HIS4::LEU2-(BamH1; +ori)/his4-X::LEU2-(NgoMIV; +ori)-URA3 MLH1-710-HA6-713::KanMX/'' MER3-His6-Flag3::NatMX/'' MLH2-Myc13::HphMX/”*

VBD1630 *a/l ho::hisG/" leu2::hisG/’’ ura3/'' HIS4::LEU2-(BamH1; +ori)/his4-X::LEU2-(NgoMIV; +ori)-URA3 MLH1-710-HA6-713::KanMX/'' MLH2-Myc13::HphMX/”*

VBD1631 *a/l ho::hisG/" leu2::hisG/’’ ura3/'' HIS4::LEU2-(BamH1; +ori)/his4-X::LEU2-(NgoMIV; +ori)-URA3 mlh2∆::HphMX/”*

VBD1635 *a/l ho::hisG/" leu2::hisG/’’ ura3/'' HIS4::LEU2-(BamH1; +ori)/his4-X::LEU2-(NgoMIV; +ori)-URA3 MER3-R893E-His6-Flag3::NatMX/''*

VBD1637 *a/l ho::hisG/" leu2::hisG/’’ ura3/'' HIS4::LEU2-(BamH1; +ori)/his4-X::LEU2-(NgoMIV; +ori)-URA3 MER3-R893E-His6-Flag3::NatMX/'' MLH2-Myc13::HphMX/”*

VBD1649 *a/l ho::hisG/" leu2::hisG/’’ ura3/'' HIS4::LEU2-(BamH1; +ori)/his4-X::LEU2-(NgoMIV; +ori)-URA3 MLH2-Myc13::HphMX/” zip4∆ ::HphMX/”*

VBD1653 *a/l ho::hisG/" leu2::hisG/’’ ura3/'' HIS4::LEU2-(BamH1; +ori)/his4-X::LEU2-(NgoMIV; +ori)-URA3 MER3-R893E-His6-Flag3::NatMX/'' zip4∆::HphMX/”*

VBD1670 *a/l ho::hisG/" leu2::hisG/’’ ura3/'' HIS4::LEU2-(BamH1; +ori)/his4-X::LEU2-(NgoMIV; +ori)-URA3 MER3-His6-Flag3::NatMX/'' MLH2-Myc13::HphMX/”*

VBD1676 *a/l ho::hisG/" leu2::hisG/’’ ura3/'' HIS4::LEU2-(BamH1; +ori)/his4-X::LEU2-(NgoMIV; +ori)-URA3 msh4∆::HphMX/”*

VBD1681 *a/l ho::hisG/" leu2::hisG/’’ ura3/'' HIS4::LEU2-(BamH1; +ori)/his4-X::LEU2-(NgoMIV; +ori)-URA3 MLH1-710-HA6-713::KanMX/'' MER3-R893E-His6-Flag3::NatMX/'' MLH2-Myc13::HphMX/”*

VBD1682 *a/l ho::hisG/" leu2::hisG/’’ ura3/'' HIS4::LEU2-(BamH1; +ori)/his4-X::LEU2-(NgoMIV; +ori)-URA3 mlh2∆::HphMX/” msh4∆::HphMX/”*

VBD1684 *a/l ho::hisG/" leu2::hisG/’’ ura3/'' HIS4::LEU2-(BamH1; +ori)/his4-X::LEU2-(NgoMIV; +ori)-URA3 MER3-R893E-His6-Flag3::NatMX/'' msh4∆::HphMX/”*

VBD1702 *a/l ho::hisG/" leu2::hisG/’’ ura3/'' HIS4::LEU2-(BamH1; +ori)/his4-X::LEU2-(NgoMIV; +ori)-URA3 MLH2-Myc13::HphMX/” spo11∆::HphMX/”*

VBD1704 *a/l ho::hisG/" leu2::hisG/’’ ura3/'' HIS4::LEU2-(BamH1; +ori)/his4-X::LEU2-(NgoMIV; +ori)-URA3 MLH2-Myc13::HphMX/” msh2∆::HphMX/”*

VBD1706 *a/l ho::hisG/" leu2::hisG/’’ ura3/'' HIS4::LEU2-(BamH1; +ori)/his4-X::LEU2-(****BamH1****; +ori)-URA3 MER3-R893E-His6-Flag3::NatMX/'' MLH2-Myc13::HphMX/”*

VBD1707 *a/l ho::hisG/" leu2::hisG/’’ ura3/'' HIS4::LEU2-(BamH1; +ori)/his4-X::LEU2-(****BamH1****; +ori)-URA3 MLH2-Myc13::HphMX/”*

VBD1710 *a/l ho::hisG/" leu2::hisG/’’ ura3/'' HIS4::LEU2-(BamH1; +ori)/his4-X::LEU2-(****BamH1****; +ori)-URA3 MER3-His6-Flag3::NatMX/'' MLH2-Myc13::HphMX/”*

VBD1714 *a/l ho::hisG/" leu2::hisG/’’ ura3/'' HIS4::LEU2-(BamH1; +ori)/his4-X::LEU2-(NgoMIV; +ori)-URA3 zip4∆::KanMX/” mer3∆::HphMX/” mlh2∆ ::NatMX/”*

VBD1726 *a/l ho::hisG/" leu2::hisG/’’ ura3/'' HIS4::LEU2-(BamH1; +ori)/his4-X::LEU2-(NgoMIV; +ori)-URA3 zip4∆::KanMX/” mer3∆::HphMX/”*

VBD1750 *a/l ho::hisG/" leu2::hisG/’’ ura3/'' HIS4::LEU2-(BamH1; +ori)/his4-X::LEU2-(NgoMIV; +ori)-URA3 MER3-K167A-His6-Flag3::NatMX/''*

VBD1756 *a/l ho::hisG/" leu2::hisG/’’ ura3/'' HIS4::LEU2-(BamH1; +ori)/his4-X::LEU2-(NgoMIV; +ori)-URA3 MER3-K167A-His6-Flag3::NatMX/'' mlh2∆::HphMX/”*

VBD1757 *a/l ho::hisG/" leu2::hisG/’’ ura3/'' HIS4::LEU2-(BamH1; +ori)/his4-X::LEU2-(NgoMIV; +ori)-URA3 MER3-K167A-His6-Flag3::NatMX/'' msh4∆::HphMX/”*

VBD1758 *a/l ho::hisG/" leu2::hisG/’’ ura3/'' HIS4::LEU2-(BamH1; +ori)/his4-X::LEU2-(NgoMIV; +ori)-URA3 MER3-K167A-His6-Flag3::NatMX/'' msh4∆::HphMX/” mlh2∆::HphMX/”*

VBD1794 *a/l ho::hisG/" leu2::hisG/’’ ura3/'' HIS4::LEU2-(BamH1; +ori)/his4-X::LEU2-(NgoMIV; +ori)-URA3 MER3-His6-Flag3::NatMX/'' spo11∆::HphMX/”*

VBD1795 *a/l ho::hisG/" leu2::hisG/’’ ura3/'' HIS4::LEU2-(BamH1; +ori)/his4-X::LEU2-(NgoMIV; +ori)-URA3 MER3-R893E-His6-Flag3::NatMX/'' spo11∆::HphMX/”*

VBD1796 *a/l ho::hisG/" leu2::hisG/’’ ura3/'' HIS4::LEU2-(BamH1; +ori)/his4-X::LEU2-(NgoMIV; +ori)-URA3 mlh2∆::HphMX/” spo11∆::HphMX/”*

VBD-HY1 BLY107 (S288c)/BLY114 (SK1) *msh2∆::HphMX/”*  from Martini *et al.* 2011

VBD-HY2 VBD-HY1 *mlh2∆::KanMX/”*

VBD-HY3 VBD-HY1 *MER3-R893E/''*

VBD-HY4 VBD-HY1 *MER3-K167A-His6-Flag3::NatMX/''*

VBD-HY5 VBD-HY1 *MER3-K167A-His6-Flag3::NatMX/'' mlh2∆::KanMX/”*

VBD-HY6 SK1/S288C hybrid

*a/l ho::LYS2/ho ura3/URA3 leu2::hisG/LEU2 lys2/LYS2 arg4/ARG4 thr1-A/THR1 ADE8/ade8*

VBD-HY7 VBH-HY6 *mlh2∆::KanMX/”*

Haploids

**Strain name Genotype**

VBH854 *MATl ade5-1 lys2::InsE-A14 trp1-289 his7-2 leu2-3,112 ura3-52*

VBH865 *MATl ade5-1 lys2::InsE-A14 trp1-289 his7-2 leu2-3,112 ura3-52 mlh1∆::HphMX*

VBH868 *MATl ade5-1 lys2::InsE-A14 trp1-289 his7-2 leu2-3,112 ura3-52 MLH1-710-His6-Flag3-713::KanMX*

VBH1226 *MATl ade5-1 lys2::InsE-A14 trp1-289 his7-2 leu2-3,112 ura3-52 MLH1-710-HA6-713::KanMX*
