# Supplementary material for: Concerted action of the MutLβ heterodimer and Mer3 helicase regulates the global extent of meiotic gene conversion
Source: eLife. 2017 Jan 4;6:e21900. doi: 10.7554/eLife.21900 (PMC5215242; doi:10.7554/eLife.21900)
Supplement: Supplementary file 3. — DOI: http://dx.doi.org/10.7554/eLife.21900.023 [file elife-21900-supp3.docx]

**Supplementary File 3: Strains used for each figure panel**

**Strain name Figure panel**

VBD1082 *Fig. 7c, Supplementary File 1*

VBD1311 *Fig. 2c, 2d, 3a, 7a, 7b, Fig. 3-Fig. supplement 1d, Fig. 7-Fig. supplement 1, Supplementary File 1*

VBD1382 *Fig. 7a*

VBD1337 *Fig. 2c, 2d, 3a*

VBD1414 *Fig. 7a, 7b, Supplementary File 1*

VBD1420 *Fig. 3b, 5a, 7a, 7^e^, Fig3-Fg. Supplement 1d*

VBD1454 *Fig. 3b, 3d, Fig. 3-Fig. supplement 1c*

VBD1456 *Fig. 2c, 2d, 3b, 3d*

VBD1490 *Fig. 3d*

VBD1494 *Fig. 2c, 2d*

VBD1550 *Fig. 3d*

VBD1552 *Fig. 3d*

VBD1564 *Fig. 3-Fig. supplement 1c*

VBD1576 *Fig. 3-Fig. supplement 1c*

VBD1579 *Fig. 3-Fig. supplement 1c*

VBD1602 *Fig. 7c, Supplementary File 1*

VBD1604 *Supplementary File 1*

VBD1628 *Fig. 5a*

VBD1629 *Fig. 5b*

VBD1630 *Fig. 5e*

VBD1631 *Fig. 7a, 7b, Supplementary File 1*

VBD1635 *Fig. 7a, 7b, Supplementary File 1*

VBD1637 *Fig. 5c*

VBD1649 *Supplementary File 1*

VBD1653 *Fig. 7c, Supplementary File 1*

VBD1670 *Fig. 5a, 5c, 5f*

VBD1676 *Fig. 7c, Supplementary File 1*

VBD1681 *Fig. 5b*

VBD1682 *Fig. 7c, Supplementary File 1*

VBD1684 *Fig. 7c, Supplementary File 1*

VBD1702 *Fig. 5c*

VBD1704 *Fig. 5-Fig. supplement 1*

VBD1706 *Fig. 5f*

VBD1707 *Fig. 5-Fig. supplement 1*

VBD1710 *Fig. 5f*

VBD1714 *Supplementary File 1*

VBD1726 *Supplementary File 1*

VBD1750 *Fig. 7b, 7e, Supplementary File 1*

VBD1756 *Supplementary File 1*

VBD1757 *Fig. 7c, Supplementary File 1*

VBD1758 *Fig. 7c, Supplementary File 1*

VBD1794 *Fig. 7a*

VBD1795 *Fig. 7a*

VBD1796 *Fig. 7a*

VBD-HY1 *Fig. 7b, 8c, 8d, Fig. 8-Fig. supplement 1a, b, c and d, Supplementary File 1*

VBD-HY2 *Fig. 8c, 8d, Fig. 8-Fig. supplement 1a, b, c and d, Supplementary File 1*

VBD-HY3 *Fig. 7b, 8c, 8d, Fig. 8-Fig. supplement 1a, b, c and d, Supplementary File 1*

VBD-HY4 *Fig. 8c, 8d, Fig. 8-Fig. supplement 1a, b and c, Supplementary File 1*

VBD-HY5 *Fig. 8c, 8d, Fig. 8-Fig. supplement 1a and c, Supplementary File 1*

VBD-HY6 *Fig. 7b, Supplementary File 1*

VBD-HY7 *Fig. 7b, Supplementary File 1*

VBH854 *Fig. 2b*

VBH865 *Fig. 2b*

VBH868 *Fig. 2b*

VBH1226 *Fig. 2b*
